# Are duplicated genes responsible for anthracnose resistance in common bean?

**DOI:** 10.1371/journal.pone.0173789

**Published:** 2017-03-15

**Authors:** Larissa Carvalho Costa, Rafael Storto Nalin, Magno Antonio Patto Ramalho, Elaine Aparecida de Souza

**Affiliations:** 1 Department of Biology, Universidade Federal de Lavras, Lavras, Minas Gerais, Brazil; 2 Department of Genetics, Escola Superior de Agricultura Luiz de Queiroz/Universidade de São Paulo, Piracicaba, São Paulo, Brazil; Universita degli Studi di Pisa, ITALY

## Abstract

The race 65 of *Colletotrichum lindemuthianum*, etiologic agent of anthracnose in common bean, is distributed worldwide, having great importance in breeding programs for anthracnose resistance. Several resistance alleles have been identified promoting resistance to this race. However, the variability that has been detected within race has made it difficult to obtain cultivars with durable resistance, because cultivars may have different reactions to each strain of race 65. Thus, this work aimed at studying the resistance inheritance of common bean lines to different strains of *C*. *lindemuthianum*, race 65. We used six *C*. *lindemuthianum* strains previously characterized as belonging to the race 65 through the international set of differential cultivars of anthracnose and nine commercial cultivars, adapted to the Brazilian growing conditions and with potential ability to discriminate the variability within this race. To obtain information on the resistance inheritance related to nine commercial cultivars to six strains of race 65, these cultivars were crossed two by two in all possible combinations, resulting in 36 hybrids. Segregation in the F_2_ generations revealed that the resistance to each strain is conditioned by two independent genes with the same function, suggesting that they are duplicated genes, where the dominant allele promotes resistance. These results indicate that the specificity between host resistance genes and pathogen avirulence genes is not limited to races, it also occurs within strains of the same race. Further research may be carried out in order to establish if the alleles identified in these cultivars are different from those described in the literature.

## Introduction

Common bean (*Phaseolus vulgaris* L.) is widely cultivated, especially in developing countries. Its grains are excellent source of proteins and minerals. Great amounts of carbohydrates, iron and an essential amino acid called lysine are provided by common bean [1]. However, several factors limit the yield of common beans, such as the occurrence of plant pathogens, which cause considerable damage to the crop, leading to yield reduction [2]. Anthracnose is one of the most important diseases of common bean. It is caused by a fungus, *Colletotrichum lindemuthianum*, and can be found in every continent where common beans are cultivated, predominantly in tropical and subtropical regions, where the temperatures are mild and humidity is high [3,4]. Damages caused by anthracnose affect the quality of grains and pods. Losses may reach 100% when the seeds are infected and/or the susceptible cultivars are under favorable conditions for the pathogen to develop [2,5].

Anthracnose control must be preventive, integrating cultural, chemical and genetic measures. Resistant cultivars is the most efficacious and low-cost method to control anthracnose [6]. Nevertheless, the obtainment of cultivars with durable resistance is difficult, since there is a great pathogenic variability of the fungus, which is evidenced by the large amount of races [7–9]. It may be explained by mutation as well as other mechanisms, such as sexual and parasexual reproduction, transposable elements, fusion of hyphae [10], and also Conidial Anastomosis Tubes–CATs [11,12].

The inheritance of resistance to anthracnose has been studied extensively. Researchers identified, among different genes, several alleles related to resistance with independent effect: *Co*-1 [13], *Co*-2 [14], *Co*-3 [15], *Co*-4, *Co*-5 [16], *Co-6* [17,18], *Co*-7/*Co*-3 [19,20], *co*-8 [17], *C*o-9/*Co*-3^3^ [21,22], *Co*-10/*Co*-3^4^ [23,24], *Co*-11 [25], *Co*-12 [26], *Co*-13 [27], *Co*-14 [28], *Co*-15 [29], *Co*-16 [24], *Co*-17 [30], *Co*-u, *Co*-v, *Co*-w, *Co*-x [31], *Co*-y e *Co*-z [21]. Such findings evidence the employment of genetic resistance as an effective measure to control the disease. Multiple allelism has been found in the following loci: *Co*-1, *Co*-3, *Co*-4 e *Co*-5. Locus *Co*-1 has an allelic series, which is formed by four other alleles (*Co*-1^2^, *Co*-1^3^, *Co*-1^4^ e *Co*-1^5^) [32–34]. Locus *Co*-3 contains the allele *Co*-3^2^, [35], whereas locus *Co*-4 contains two alleles, *Co*-4^2^ [19,36] and *Co*-4^3^, [37]. Finally, locus *Co*-5 contains the allele *Co*-5^2^ [38]. The mapping of some of these genes has revealed that they are organized in complex clusters where closely linked loci confer resistance to specific races [6,31,39,40].

Nevertheless the variability within races of *C*. *lindemuthianum* constitutes a challenge to obtain durable resistance to anthracnose [41–44]. Significant differences in the pathogenicity of six *C*. *lindemuthianum* strains, race 65, was observed when these strains were inoculated in seven common bean commercial cultivars [44]. Resistance [37] and susceptibility [34] was verified in the cultivar BAT 93 to race 65. Variability within the race also has been reported for races 73, 81 and 89 [6,43,45]. It indicates the existence of races which are not able to be discriminated by using the universal set of 12 differential cultivars [46].

In order to overcome this problem, it was proposed an alternative group of cultivars that are able to discriminate variability within race 65 of *C*. *lindemuthianum* [47]. Race 65 has been reported as a stable race. It is widely distributed in around the world, therefore, constitutes an important issue for common bean plant breeding programs, which aim anthracnose resistance [40,47,48]. This alternative differential series of cultivars allows breeders to identify the variability, not only within race 65, but also within race 81 of *C*. *lindemuthianum* [45]. These cultivars are adapted to Brazilian environmental conditions and they are commercially available, which favors their use. The adoption of this complementary system could improve discrimination of the existent variability within important races worldwide. It could also allow researchers to choose the most virulent strains during evaluation, in order to achieve durable resistance [47].

However there is no information, in the literature, about what genes of these cultivars confer resistance to different strains of race 65. In addition, it is important to verify if genetic resistance, in this case, follows interaction model gene-for-gene [49,50], as it has been verified for specific race resistance [6]. Therefore, the purpose of this work was to study the inheritance of common bean resistance to different strains of race 65 of *C*. *lindemuthianum*. The differential series of cultivars for race 65 [47] was used in this study. This information may aid breeders to choose the most suitable strategies to achieve anthracnose resistance. It also contributes to increase stability and durability of resistance in lines of common bean and to advance the knowledge about resistance genes to *C*. *lindemuthianum*.

## Materials and methods

Experiments were performed in the Laboratory of Plant Resistance to Diseases, as well as in the greenhouse, in the mist chamber, and in the experimental field of the Biology Department of the Universidade Federal de Lavras, Minas Gerais, Brazil.

### Common-bean cultivars and strains of race 65 of *C*. *lindemuthianum* used in this work

Firstly, one plant of each cultivar listed in differential cultivars for race 65 of *C*. *lindemuthianum* [47] was selected individually, in order to ensure genetic uniformity. The 12 cultivars are BRS Estilo, Majestoso, Ouro Negro, BRSMG União, BRS Valente, Ouro Vermelho, Madrepérola, Pérola, BRS Cometa, BRS Esplendor, BRSMG Talismã and BRS Supremo. Subsequently, to obtain seeds quantity enough of each cultivar, these were sown in pots containing soil and substrate Rohrbacher^®^ in the 2:1 ratio respectively. The pots were kept in a greenhouse until the time of harvest. Fertilizations followed the crop-specific recommendations.

Seven strains of race 65 of *C*. *lindemuthianum* (Cl 1614, Cl 1532, Cl1610, Cl 1740, LV 134, LV 238 and LV 145), from different regions in Brazil, were inoculated separately in the standard set of 12 differential cultivars [46]. Nine seeds of each differential cultivar was sown in plastic trays with individual cells filled with the substrate Rohrbacher^®^. Two trays were used per strain. The inoculations were carried out in the same way as were performed for evaluation of plants of F_1_ and F_2_ generations and it is described in detail in the following sections. It made possible to verify the classification of these strains as race 65. Once the race was confirmed, they were inoculated in the differential series of cultivars for race 65 [47]. Hence the existence of pathogenic variability among the strains could be elucidated.

### Diallel crosses

The cultivars seeds were sown in pots and these were kept in greenhouse under controlled environmental conditions. During the flowering period, manual crosses were performed among all cultivars two by two, in all possible combinations, which resulted in 36 hybrids F_1_. The emasculation of the floral bud and controlled pollination were performed with tweezers of fine tips. The crosses were duly identified with pieces of colored wool. Part of F_1_ generation seeds of each hybrid was sown separately under field conditions to obtain at the end of the cycle the F_2_ segregating populations seeds. The other part was used for inoculation and evaluation of F_1_ plants to reaction to the six strains of *C*. *lindemuthianum*.

### Inoculation and evaluation of F_1_ and F_2_ populations from each cross

F_1_ plants from each hybrid were evaluated to reaction to each *C*. *lindemuthianum* strain in three replications being the plot constituted by one F_1_ plant. Sowing was carried out in plastic trays with individual cells filled with the substrate Rohrbacher^®^.

F_2_ segregating populations were evaluated by using 55 seeds from each cross for each one of the *C*. *lindemuthianum* strains used. This number of seeds is sufficient to achieve a 95% significance level to find at least one individual with recessive genotype (susceptible), considering the occurrence of two genes. Sowing was carried out in plastic trays with individual cells. Cultivar Pérola was used as susceptible control in all inoculations.

The strains of *C*. *lindemuthianum* were grown on Petri dishes containing M3 culture medium[51]. Posteriorly, aiming to obtain high sporulation rates, small pieces of mycelium were transferred to sterile common-bean pods placed in test tubes for an incubation period of 10–15 days at 22°C, in the dark. Subsequently, the conidia suspensions (1.2 x 10^6^ conidia.ml^-1^) were inoculated in the primary fully expanded leaves by spraying both leaf surfaces and the stem. After inoculations, plants were kept in the greenhouse with 95% relative humidity, at 24°C for 10 days. Severity of the disease was visually evaluated according to the descriptive scale (1 to 9) [52]. Plants were classified either as resistant (1 to 3) or susceptible (4 to 9).

### Statistical analyses

Segregation hypotheses for F_2_ generation, concerning resistant and susceptible individuals, were formulated based on the observed frequencies of the phenotypes. Such hypotheses were tested by using chi-square test (*χ*^2^) and the aid of the software GENES [53]. The significance level was 5%.

## Results

### Evaluation of pathogenic variability within strains of race 65 of *C*. *lindemuthianum*

The inoculations of the seven *C*. *lindemuthianum* strains in the standard set of differential cultivars proposed by Pastor-Corrales [46] confirmed all the tested strains belonged to race 65 of *C*. *lindemuthianum*. In fact, after all inoculations, there were always two susceptible cultivars: Michelite (2^0^) and Mexico 222 (2^6^), (2^0^ + 2^6^ = 65). However, the inoculations of these same *C*. *lindemuthianum* strains in the differential series of cultivars for race 65 [47] showed differences in the spectrum of virulence among these strains (Table 1). The exceptions were the strains LV 145 and LV 238. Due to this fact, the strain LV 145 was discarded. Seven cultivars showed susceptibility to LV 134. LV 238 also caused susceptibility response in seven cultivars, but different ones. The strains Cl 1532 and Cl 1614 were pathogenic to different combinations of five cultivars each. Two cultivars were susceptible to strain Cl 1740 and one cultivar was susceptible to Cl 1610.

**Table 1:** Reaction pattern of the 12 commercial cultivars of common bean inoculated with each evaluated strain of race 65 of *C*. *lindemuthianum*.

Strains	Cultivars^a^								Classification^b^
	1,2	3,4,5	6,7	8	9	10	11	12	
Cl1532	S^c^	R^d^	S	R	S	R	R	R	65.152
Cl1610	R	R	S	R	S	R	R	S	65.168
Cl1614	R	R	S	R	S	S	S	R	65.201
Cl1740	R	R	S	R	S	R	R	R	65.136
LV238/ LV145	S	R	S	S	S	S	R	R	65.218
LV134	R	S	S	S	R	R	S	R	65.135

^a^1-Valente; 2-Ouro vermelho; 3-Ouro Negro; 4-Cometa; 5-Supremo; 6-Pérola; 7-Talismã; 8- Majestoso; 9-União; 10-Madrepérola; 11-Estilo; 12-Esplendor.

^b^Binary system used in strains classification: Estilo (2^0^), Majestoso (2^1^), Supremo (2^2^), União (2^3^), Valente (2^4^), Esplendor (2^5^), Madrepérola (2^6^), Talismã (2^7^). Adapted of Ishikawa et al. (2011).

^c^Susceptible cultivar (S).

^d^ Resistant cultivar (R).

In general, the response to the six strains differed from cultivar to cultivar. However, the cultivars, Ouro Vermelho and Valente, had the same response, as well as the cultivars, Ouro Negro, Cometa and Supremo. Pérola and Talismã cultivars were susceptible to all strains. União cultivar was resistant to only one of the six strains. The cultivars, Valente, Estilo, Madrepérola and Majestoso were susceptible to two distinct strains each. The cultivars, Esplendor, Ouro Negro, Cometa and Supremo presented the highest level of resistance, because they were susceptible to only one strain each. None was resistant to all strains.

According to the pathogenicity pattern in the different cultivars, the strains were reclassified by using the binary system, adapted from [47] (Table 1). Once the cultivars Talismã and Pérola were susceptible in all cases, they do not contain resistance genes to the tested strains. In addition, for every strain there was always at least one susceptible cultivar to it, besides Talismã and Pérola (Table 1). Thus, these two cultivars were not included in both the study of the inheritance and the allelism tests. Although the cultivars, Ouro Vermelho/Valente and Ouro Negro/Cometa/Supremo presented the same response pattern to six strains used, there could be differences related to the gene, which is associated with resistance to particular strains of race 65 of *C*. *lindemuthianum*. However due to the large number of crossings, Supremo cultivar was also not used in this study.

Therefore, in order to study the inheritance and perform the allelism test, six strains (Cl 1614, Cl 1532, Cl1610, Cl 1740, LV 134 and LV 238) were selected. In addition nine cultivars (BRS Estilo, Majestoso, Ouro Negro, BRSMG União, BRS Valente, Ouro Vermelho, Madrepérola, BRS Cometa and BRS Esplendor) were selected according to the response of the cultivar to each strain.

### Inheritance of resistance and allelism test for the strain Cl 1740 of race 65.136 of *C*. *lindemuthianum*

Strain Cl 1740 was the least pathogenic among all the six tested strains. Cultivar União was the only susceptible cultivar to it. This way, in order to understand the inheritance of resistance to strain Cl 1740, it is necessary to observe the result of the crossing between União and every other resistant cultivar in this study.

Table A in S1 File shows the response of plants (F_1_ and F_2_ generations) after inoculation with this strain. All plants in F_1_ generation, derived from crossing involving cultivar União, were resistant. Thus, resistance is conferred by a dominant allele from one or more genes.

In F_2_ generation, there were segregations which fitted the ratio 15R:1S. It was observed in plants derived from crossings between União x Valente and União x Esplendor. This event indicates these two cultivars have two independent genes with dominant alleles conferring resistance to this strain. On the other hand, it was observed a ratio of 3R:1S in the crossings involving cultivar União and the other resistant cultivars. It indicates that all the genitors, in this case, differed in one gene. Moreover the allele is dominant and responsible for conferring resistance to strain Cl 1740. There was no segregation in F_2_ populations which derived from crossing between two resistant cultivars. This could indicate they have either the same resistance alleles or different resistance alleles from the same gene.

### Inheritance of resistance and allelism test for the strain Cl 1610 of race 65.168 of *C*. *lindemuthianum*

The cultivars Esplendor and União were susceptible to strain Cl 1610. The segregation in F_2_ generations presented a ratio of 15R:1S for the crossings of these two cultivars with Valente, Madrepérola and Majestoso. Thus two genes with dominant alleles confer resistance to this strain. The segregation observed in all crossings, involving Cometa, Estilo, Ouro Negro and Ouro Vermelho with any susceptible cultivar, fitted the ratio of 3R:1S (Table B in S1 File). Therefore these cultivars differ in only one gene from the susceptible one. Besides the allele is dominant and responsible for resistance.

No segregation was observed in F_2_ generations derived from crossings between two resistant cultivars. It demonstrates that resistance to strain Cl 1610 is conferred by either a single allele of resistance or different alleles from the same gene.

The findings from the evaluation of F_1_ generation plants support the results obtained from F_2_ generation plants.

### Inheritance of resistance and allelism test for the strain LV 134 of race 65.135 of *C*. *lindemuthianum*

Five cultivars were resistant to this strain: Valente, Madrepérola, União, Ouro Vermelho and Esplendor. The result of crossing between Esplendor and any other susceptible cultivar fitted a ratio of 15R:1S in F_2_ generation. In other words, this cultivar has two independent genes with dominant alleles conferring resistance to this strain. For the other resistant cultivars, the study of the inheritance indicated that a single gene is responsible for differing resistant from susceptible cultivars, since chi-square test was not significant for the expected frequency (3R:1S) (Table C in S1 File).

The allelism test showed there was no segregation in F_2_ populations, derived from two resistant cultivars. Therefore resistance to each cultivar is defined by either a single resistance allele or different resistance alleles from the same gene.

The findings on F_1_ generation and F_2_ generation support each other.

### Inheritance of resistance and allelism test for the strain Cl 1614 of race 65.201 of *C*. *lindemuthianum*

Six cultivars were resistant to this strain: Valente, Cometa, Ouro Vermelho, Ouro Negro, Majestoso and Esplendor. There was no segregation in any crossings, two by two, involving the cultivars Valente, Cometa, Ouro Vermelho and Ouro Negro. The absence of segregation was also verified in the crossing between cultivars Majestoso and Esplendor. Hence, they have either a single resistance allele to strain Cl 1614 or different resistance alleles from the same gene. Nonetheless, segregation in a 15R:1S ratio was observed in the crossings between any resistant cultivar and one of these cultivars (Majestoso or Esplendor). This fact is an evidence that the gene related to resistance in these two cultivars is different from the genes related to resistance in the other cultivars (Valente, Cometa, Ouro Vermelho and Ouro Negro) (Table D in S1 File).

Crossings between one resistant and one susceptible cultivar fitted the ratio 3R:1S. Thus the resistant cultivars differ from the susceptible ones in only one gene. Besides the dominant allele is responsible for resistance.

Observed results in F_1_ generation is in agreement with the observed segregation in F_2_ generation.

### Inheritance of resistance and allelism test for the strain Cl 1532 of race 65.152 of *C*. *lindemuthianum*

The strain Cl 1532 was not pathogenic to six cultivars: Esplendor, Ouro Negro, Estilo, Cometa, Madrepérola and Majestoso. There was no segregation in F_2_ generation in all the crossings involving the cultivars Cometa, Estilo, Ouro Negro and Esplendor. F_2_ generation, derived from crossing between Madrepérola and Majestoso, was also completely resistant. However it was observed a segregation ratio of 15R:1S when Madrepérola and Majestoso were crossed with the other resistant cultivars. Hence these two cultivars have a resistance gene to strain Cl 1532 different from the resistance gene found in the cultivars Cometa, Estilo, Ouro Negro and Esplendor (Table E in S1 File).

The resistance inheritance study for these cultivars confirmed that they differ from the susceptible ones in only one gene, since the observed segregations fitted a ratio of 3R:1S in all crossings between a resistant and a susceptible cultivar. It also indicates that the dominant allele is the responsible for resistance.

The observed results in F_1_ generation plants support the results obtained in F_2_ generation plants.

### Inheritance of resistance and allelism test for the strain LV 238 of race 65.218 of *C*. *lindemuthianum*

Four cultivars were resistant to this strain: Esplendor, Ouro Negro, Estilo and Cometa. There was no segregation in F_2_ generations derived from crossings, two by two, involving Cometa, Estilo and Esplendor. Nevertheless, whenever they were crossed with the cultivar Ouro Negro, a segregation ratio of 15R:1S was observed. This fact indicates that cultivar Ouro Negro has a different resistance gene to strain LV 238 comparing to the cultivars Cometa, Estilo and Esplendor (Table F in S1 File).

The resistance inheritance study for these cultivars, regarding strain LV 238, confirmed the previous results, since it was observed a segregation ratio of 3R:1S in all crossings involving a resistant and a susceptible cultivar. Thus resistant cultivars differ from the susceptible ones in only one gene. Moreover the dominant allele is responsible for resistance.

As well as in other cases the findings from the evaluation of F_1_ generation plants support the results obtained in F_2_ generation plants.

### Genotype of the evaluated cultivars and number of genes related to resistance to the six strains

The resistance of the cultivars to each inoculated strain is independent. It means that different genes are associated with resistance to the six strains of race 65 of *C*. *lindemuthianum* tested in this study. It may be verified by the different results found in F_2_ generations from all crossings combined with the inoculation of different strains. Furthermore, for each strain, there were segregations in F_2_ generations that fitted a ratio of 15R:1S. It suggests the presence of duplicate genes, which confer resistance to a particular strain. Therefore, a total of 12 genes is involved in the resistance to the six strains. Based in the results of Tables A to F in S1 File, the possible genotypes were established, considering the resistance genes to the six strains of race 65 of *C*. *lindemuthianum* as well as each one of the nine evaluated cultivars (Table 2).

**Table 2:** Genotypes and phenotypes of the nine cultivars for the resistance genes to each strain of race 65 of *C*. *lindemuthianum*.

Cultivars	Resistance genes					
	Cl 1740	Cl1610	LV 134	Cl1614	Cl 1532	LV 238
União	*co*_A_*co*_A_*co*_B_*co*_B_^1^	*co*_C_*co*_C_*co*_D_*co*_D_	*co*_E_*co*_E_*Co*_F_*Co*_F_	*co*_G_*co*_G_*co*_H_*co*_H_	*co*_I_*co*_I_*co*_J_*co*_J_	*co*_K_*co*_K_*co*_L_*co*_L_
	(Susceptible)^2^	(Susceptible)	(Resistant)	(Susceptible)	(Susceptible)	(Susceptible)
Majestoso	*Co*_A_*Co*_A_*co*_B_*co*_B_	*Co*_C_*Co*_C_*Co*_D_*Co*_D_	*co*_E_*co*_E_*co*_F_*co*_F_	*Co*_G_*Co*_G_*co*_H_*co*_H_	*Co*_I_*Co*_I_*co*_J_*co*_J_	*co*_K_*co*_K_*co*_L_*co*_L_
	(Resistant)	(Resistant)	(Susceptible)	(Resistant)	(Resistant)	(Susceptible)
Madrepérola	*Co*_A_*Co*_A_*co*_B_*co*_B_	*Co*_C_*Co*_C_*Co*_D_*Co*_D_	*co*_E_*co*_E_*Co*_F_*Co*_F_	*co*_G_*co*_G_*co*_H_*co*_H_	*Co*_I_*Co*_I_*co*_J_*co*_J_	*co*_K_*co*_K_*co*_L_*co*_L_
	(Resistant)	(Resistant)	(Resistant)	(Susceptible)	(Resistant)	(Susceptible)
Valente	*Co*_A_*Co*_A_*Co*_B_*Co*_B_	*Co*_C_*Co*_C_*Co*_D_*Co*_D_	*co*_E_*co*_E_*Co*_F_*Co*_F_	*co*_G_*co*_G_*Co*_H_*Co*_H_	*co*_I_*co*_I_*co*_J_*co*_J_	*co*_K_*co*_K_*co*_L_*co*_L_
	(Resistant)	(Resistant)	(Resistant)	(Resistant)	(Susceptible)	(Susceptible)
Ouro vermelho	*Co*_A_*Co*_A_*co*_B_*co*_B_	*Co*_C_*Co*_C_*Co*_D_*Co*_D_	*co*_E_*co*_E_*Co*_F_*Co*_F_	*co*_G_*co*_G_*Co*_H_*Co*_H_	*co*_I_*co*_I_*co*_J_*co*_J_	*co*_K_*co*_K_*co*_L_*co*_L_
	(Resistant)	(Resistant)	(Resistant)	(Resistant)	(Susceptible)	(Susceptible)
Estilo	*Co*_A_*Co*_A_*co*_B_*co*_B_	*co*_C_*co*_C_*Co*_D_*Co*_D_	*co*_E_*co*_E_*co*_F_*co*_F_	*co*_G_*co*_G_*co*_H_*co*_H_	*co*_I_*co*_I_*Co*_J_*Co*_J_	*co*_K_*co*_K_*Co*_L_*Co*_L_
	(Resistant)	(Resistant)	(Susceptible)	(Susceptible)	(Resistant)	(Resistant)
Cometa	*Co*_A_*Co*_A_*co*_B_*co*_B_	*co*_C_*co*_C_*Co*_D_*Co*_D_	*co*_E_*co*_E_*co*_F_*co*_F_	*Co*_G_*Co*_G_*co*_H_*co*_H_	*co*_I_*co*_I_*Co*_J_*Co*_J_	*co*_K_*co*_K_*Co*_L_*Co*_L_
	(Resistant)	(Resistant)	(Susceptible)	(Resistant)	(Resistant)	(Resistant)
Ouro Negro	*Co*_A_*Co*_A_*co*_B_*co*_B_	*co*_C_*co*_C_*Co*_D_*Co*_D_	*co*_E_*co*_E_*co*_F_*co*_F_	*Co*_G_*Co*_G_*co*_H_*co*_H_	*co*_I_*co*_I_*Co*_J_*Co*_J_	*Co*_K_*Co*_K_*co*_L_*co*_L_
	(Resistant)	(Resistant)	(Susceptible)	(Resistant)	(Resistant)	(Resistant)
Esplendor	*Co*_A_*Co*_A_*Co*_B_*Co*_B_	*co*_C_*co*_C_*co*_D_*co*_D_	*Co*_E_*Co*_E_*Co*_F_*Co*_F_	*Co*_G_*Co*_G_*co*_H_*co*_H_	*co*_I_*co*_I_*Co*_J_*Co*_J_	*co*_K_*co*_K_*Co*_L_*Co*_L_
	(Resistant)	(Susceptible)	(Resistant)	(Resistant)	(Resistant)	(Resistant)

^1^ *Co* symbolizes the dominant alleles and e *co* symbolizes the recessives alleles. The letters A, B, C, D, E, F, G, H, I, J, K e L symbolizes the identified resistance genes.

^2^ Phenotype of the cultivars for each strain

The cultivars Valente and Ouro Vermelho have different resistance gene to strain LV 1740, although their responses to the six evaluated strains were the same. The cultivars Ouro Negro and Cometa also presented the same pattern of response to the six strains. However, they have different genotypes for the genes that confer resistance to strain LV 238 because the segregation in F_2_ generation, derived from crossing between them, fitted a ratio of 15R:1S (Table F in S1 File).

## Discussion

Obtaining anthracnose resistant cultivars to race 65 of *C*. *lindemuthianum* is one of the main goals for common bean breeding programs. This work and previous studies [42,44] observed a large pathogenic variability within this race, which makes more difficult the development of cultivars with durable resistance. Moreover, variability within other races (as 73, 81 and 89) has been detected [6,43,54]. Thus it is important to identify resistance sources, which are able to discriminate such variability within each race. In fact, it has been done for races 65 and 81 of *C*. *lindemuthianum* [45,47].

Regional cultivars, capable of detecting pathogenic variability within the races, should complement the standard set of differential cultivars [46]. This procedure would allow a better characterization of the strains of *C*. *lindemuthianum* in different regions on the world, where common beans are cultivated.

Eight different patterns of plant responses were identified among the 12 commercial cultivars of common bean when they were inoculated with six different strains of race 65 of *C*. *lindemuthianum*. In addition, the existence of at least six different resistance genes to the six evaluated strains was expected among the cultivars, since there were six different spectrum of pathogenicity among the strains. However 12 independent genes were identified as responsible for resistance to six strains used, being two independent genes per strain. These results support the gene-for-gene theory proposed by Flor [49,50] and constitute an evidence of the presence of vertical resistance, which has already been reported for this pathosystem [44]. Other studies also emphasize that interaction between genotypes of *P*. *vulgaris* and *C*. *lindemuthianum* is highly specific [40]. Yet the present work demonstrates that this specificity can be found not only among the races, but also among the strains of the same race in a high frequency.

Host-pathogen co-evolution in gene-for-gene interaction systems is characterized by two events: Pathogen change its avirulence factors to avoid being recognized by the plant resistance proteins, whereas the hosts develop new specificities in their resistance proteins to identify the corresponding Avirulence factor [55]. There is large evidence of polymorphism of alleles in resistance and avirulence loci of plants and pathogens respectively. [56–58].

In this work, it was verified that resistance to each strain of race 65 is controlled by two independent genes, considering the evaluated cultivars. What would be the advantage in terms of evolution the presence of two independent genes performing the same function in the six cases analyzed? One hypothesis is that they are duplicated genes. In the duplicated gene of the host, one of the copies is a spare gene that could be transformed in order to recognize a different avirulence protein. Common bean is an autogamous species and its intercrossing rate is low [59,60]. Hence the presence of duplicated genes associated with resistance is an advantage.

Wheat is known by having high frequency of gene duplication in regions where recombination rates are high. Generally the resistance genes, which need to evolve quickly, are located in these regions [61,62]. In the genome of common bean, tandem duplication followed by recombination between clusters, with several resistance genes, can be observed very often [63]. These events may be involved in the evolution of the genes, which are constantly under selective pressure from the pathogen. Gene blocks, which confer resistance to several races of *C*. *lindemuthianum*, are also found in linkage groups of common bean [6,40,64].

On the other hand, *C*. *lindemuthianum* shows high variability as a result of sexual and asexual mechanisms of recombination [12,65,66]. The occurrence of anastomosis tubes among conidia (CATs) is a mechanism of asexual recombination and it is very common in *C*. *lindemuthianum*. It has been observed in different strains of race 65 [9]. CATs have probably contributed to rapid evolution of the Avirulence factors in this species. Rapid evolution of both, Resistance (host) and Avirulence (pathogen) genes may be influenced by the presence of transposable elements in the chromosomes of both species [67]. The dynamic recombination in the resistance genes clusters also can be due the presence of transposable elements. Activation of these elements is regulated by epigenetic variation, including DNA methylation and biotic and abiotic stresses [68]. Therefore, studies on common bean genome can contribute to elucidate the mechanisms, which are responsible for rapid evolution of resistance genes in this species [69].

Several alleles *Co*’s, which confer resistance to race 65, were identified in the following clusters: *Co*-1, *Co*-2, *Co*-3, *Co*-4 and *Co*-5. These clusters were mapped respectively in the following linkage groups of common bean: Pv01, Pv11, Pv04, Pv08 and Pv07 [6,40]. Thus, further studies should be carried out to verify if the 12 resistance genes, found in this study, are different from the others described in the literature. Furthermore, complementary studies must be performed to figure out the genetic architecture of resistance to these strains. One must also observe if these genes either belong to a unique cluster or are spread among several clusters in the linkage group of common bean.

The cultivars 49–242, TO, G233, PI 207.262, TU, and AB 136 have been widely used in plant breeding programs of common bean in Brazil. Each of these cultivars have resistance alleles (*Co*-1, *Co*-2, *Co*-4, *Co*-4^2^, *Co*-4^3^, *Co*-5 and *Co*-6), which confer resistance to race 65 of *C*. *lindemuthianum* among others. Common bean breeding programs incorporated these resistance genes in the cultivars, which are adapted to local conditions [70–74]. Hence there are probably some of these resistance alleles among the 12 resistance genes in the commercial cultivars evaluated in this study.

Cultivar Ouro Negro carries the resistance gene *Co*-10 [33], renamed *Co*-3^4^ [75]. It presents a wide range of resistance; however it does not confer resistance to race 65 [23,37,76]. Although, five duplicated genes associated with resistance to race 65 of *C*. *lindemuthianum*, were identified in this cultivar in the present study. It was susceptible only to strain LV134 (65.135). Cultivar Talismã is considered resistant to race 65 of *C*. *lindemuthianum* and therefore, recommended for crops in the state of Minas Gerais (Brazil) [77]. In this work, it was susceptible to all evaluated strain. Susceptibility of Talismã cultivar to certain strains of race 65 was also reported [44,78]. These results confirm that classification of a cultivar as resistant or susceptible may vary according to the inoculated strain. In the light of such pathogenic variability presented by the fungus, evaluation must be performed using more than one strain. Besides, the strains must be collected in the same geographical region that the cultivar will be cultivated.

There are two breeding strategies that are recommended for obtaining durable resistance to anthracnose. First is pyramiding of resistance genes to different races of *C*. *lindemuthianum*. This strategy may not result in durable resistance, because this procedure use only one strain from each race, in contrast, this study verified the existence of different genes associated with resistance to different strains of the same race of *C*. *lindemuthianum*. Resistance genes, used in common bean breeding programs, are unlikely to confer resistance to all strains from the same race. Moreover, gene pyramiding is a laborious and time consuming process and all efforts are concentrated into a single cultivar [73,79–81]. The second strategy is the use of multiline derived from cultivars with different resistance genes to different strains of several *C*. *lindemuthianum* races. This alternative may provide better results, because the multiline could increase resistance stability [81].

Considering the occurrence of so many genes associated with resistance to different races and different strains from the same race, a third and interesting strategy would be recurrent selection aiming anthracnose resistance. It would allow accumulating large amounts of resistance genes from different chromosomes in a single cultivar. This strategy has been used with success in common bean breeding programs that aim to obtain lines with durable resistance to *Pseudocercospora griseola* [82]. It was verified that the pathosystem common bean–*P*. *griseola* presents vertical and horizontal resistance [83]. These authors also observed that the recurrent selection has provide accumulate both types of resistance. Although none of the evaluated cultivars in this study showed resistance to all tested strains, they contain different resistance genes to race 65 of *C*. *lindemuthianum*. They also have the advantage of being adapted to local conditions and present phenotype with desirable agronomic traits. Thus they could be used in breeding programs aiming anthracnose resistance to race 65.

The findings in this work support the evidences found in several studies on pathogenic variability within the same race. It has been confirmed the existence of different genes associated with common bean resistance to different strains of race 65 of *C*. *lindemuthianum*.

## Supporting information

S1 File Table A to F presents the reaction of parents and F_1_ offspring, and expected ratio of resistant (R) and susceptible plants (S) in F_2_ generation in each cross inoculated with the strains Cl 1740, Cl 1610, LV 134, Cl 1614, Cl 1532, LV 238, respectively. Inbred lines used: V = Valente; C = Cometa; U = União; Est = Estilo; Mp = Madrepérola; OV = Ouro Vermelho; ON = Ouro Negro; Mj = Majestoso; Esp = Esplendor.
(PDF)
