# Supplementary material for: Are duplicated genes responsible for anthracnose resistance in common bean?
Source: PLoS One. 2017 Mar 15;12(3):e0173789. doi: 10.1371/journal.pone.0173789 (PMC5351970; doi:10.1371/journal.pone.0173789)
Supplement: S1 File — Inbred lines used: V = Valente; C = Cometa; U = União; Est = Estilo; Mp = Madrepérola; OV = Ouro Vermelho; ON = Ouro Negro; Mj = Majestoso; Esp = Esplendor. (PDF) [file pone.0173789.s001.pdf]

Supporting Information

Table A. Reaction of parents and F_1 offspring, and expected ratio of resistant (R) and susceptible plants (S) in F_2 generation in each cross inoculated with strain Cl 1740 (65.136).

Crosses	Reaction	Generation				χ^2	*p-value*
		F_1	F_2				
			Obs. Freq.		Expec. Freq.		
			R	S			
V × C[a]	R × R	R	54	0	1:0	-	-
V × U	R × S	R	49	5	15:1	0.83	0.36
V × Est	R × R	R	54	0	1:0	-	-
V × Mp	R × R	R	54	0	1:0	-	-
V × OV	R × R	R	53	0	1:0	-	-
V × ON	R × R	R	53	0	1:0	-	-
V × Mj	R × R	R	47	0	1:0	-	-
V × Esp	R × R	R	54	0	1:0	-	-
C × U	R × S	R	26	14	3:1	2.13	0.14
C × Est	R × R	R	50	0	1:0	-	-
C × Mp	R × R	R	54	0	1:0	-	-
C × OV	R × R	R	52	0	1:0	-	-
C × ON	R × R	R	47	0	1:0	-	-
C × Mj	R × R	R	53	0	1:0	-	-
C × Esp	R × R	R	48	0	1:0	-	-
U × Est	S × R	R	33	15	3:1	1.00	0.32
U × Mp	S × R	R	13	5	3:1	0.07	0.79
U × OV	S × R	R	41	13	3:1	0.02	0.88
U × ON	S × R	R	37	17	3:1	1.21	0.27
U × Mj	S × R	R	43	7	3:1	3.23	0.07
U × Esp	S × R	R	45	2	15:1	0.32	0.57
Est × Mp	R × R	R	52	0	1:0	-	-
Est × OV	R × R	R	52	0	1:0	-	-
Est × ON	R × R	R	54	0	1:0	-	-
Est × Mj	R × R	R	54	0	1:0	-	-
Est × Esp	R × R	R	48	0	1:0	-	-
Mp × OV	R × R	R	52	0	1:0	-	-
Mp × ON	R × R	R	47	0	1:0	-	-
Mp × Mj	R × R	R	51	0	1:0	-	-
Mp × Esp	R × R	R	34	0	1:0	-	-
OV × ON	R × R	R	54	0	1:0	-	-
OV × Mj	R × R	R	54	0	1:0	-	-
OV × Esp	R × R	R	48	0	1:0	-	-
ON × Mj	R × R	R	39	0	1:0	-	-
ON × Esp	R × R	R	47	0	1:0	-	-
Mj × Esp	R × R	R	45	0	1:0	-	-

[a]V = Valente; C = Cometa; U = União; Est = Estilo; Mp = Madrepérola; OV = Ouro Vermelho; ON = Ouro Negro; Mj = Majestoso; Esp = Esplendor

Table B. Reaction of parents and F_1 offspring, and expected ratio of resistant (R) and susceptible plants (S) in F_2 generation in each cross inoculated with strain Cl 1610 (65.168)

Crosses	Reaction	Generation				χ^2	*p- value*
			F_2				
		F_1	Obs. Freq.		Expec. Freq.		
			R	S			
V × C[a]	R × R	R	50	0	1:0	-	-
V × U	R × S	R	46	4	15:1	0.26	0.61
V × Est	R × R	R	50	0	1:0	-	-
V × Mp	R × R	R	50	0	1:0	-	-
V × OV	R × R	R	50	0	1:0	-	-
V × ON	R × R	R	50	0	1:0	-	-
V × Mj	R × R	R	50	0	1:0	-	-
V × Esp	R × S	R	45	5	15:1	1.20	0.27
C × U	R × S	R	31	14	3:1	0.90	0.34
C × Est	R × R	R	99	0	1:0	-	-
C × Mp	R × R	R	54	0	1:0	-	-
C × OV	R × R	R	53	0	1:0	-	-
C × ON	R × R	R	50	0	1:0	-	-
C × Mj	R × R	R	54	0	1:0	-	-
C × Esp	R × S	R	42	8	3:1	2.16	0.14
U × Est	S × R	R	33	16	3:1	1.53	0.22
U × Mp	S × R	R	39	5	15:1	1.96	0.16
U × OV	S × R	R	37	14	3:1	0.16	0.69
U × ON	S × R	R	37	13	3:1	0.03	0.87
U × Mj	S × R	R	45	6	15:1	2.65	0.10
U × Esp	S × S	S	0	49	0:1	-	-
Est × Mp	R × R	R	50	0	1:0	-	-
Est × OV	R × R	R	50	0	1:0	-	-
Est × ON	R × R	R	49	0	1:0	-	-
Est × Mj	R × R	R	54	0	1:0	-	-
Est × Esp	R × S	R	43	8	3:1	2.36	0.12
Mp × OV	R × R	R	54	0	1:0	-	-
Mp × ON	R × R	R	54	0	1:0	-	-
Mp × Mj	R × R	R	52	0	1:0	-	-
Mp × Esp	R × S	R	90	4	15:1	0.64	0.42
OV × ON	R × R	R	100	0	1:0	-	-
OV × Mj	R × R	R	53	0	1:0	-	-
OV × Esp	R × S	R	81	18	3:1	2.45	0.12
ON × Mj	R × R	R	50	0	1:0	-	-
ON × Esp	R × S	R	40	10	3:1	0.67	0.41
Mj × Esp	R × S	R	47	3	15:1	0.00	0.94

[a]V = Valente; C = Cometa; U = União; Est = Estilo; Mp = Madrepérola; OV = Ouro Vermelho; ON = Ouro Negro; Mj = Majestoso; Esp = Esplendor

Table C. Reaction of parents and F_1 offspring, and expected ratio of resistant (R) and susceptible plants (S) in F_2 generation in each cross inoculated with strain LV 134 (65.135)

Crosses	Reaction	Generation				χ^2	*p- value*
		F_1	F_2				
			Obs. Freq.		Expec. Freq.		
			R	S			
V × C[a]	R × S	R	40	12	3:1	0.10	0.75
V × U	R × R	R	53	0	1:0	-	-
V × Est	R × S	R	67	31	3:1	2.29	0.13
V × Mp	R × R	R	52	0	1:0	-	-
V × OV	R × R	R	50	0	1:0	-	-
V × ON	R × S	R	41	12	3:1	0.16	0.69
V × Mj	R × S	R	44	10	3:1	1.21	0.27
V × Esp	R × R	R	54	0	1:0	-	-
C × U	S × R	R	-	-	-	-	-
C × Est	S × S	S	0	54	0:1	-	-
C × Mp	S × R	R	48	8	3:1	3.43	0.06
C × OV	S × R	R	40	9	3:1	1.15	0.28
C × ON	S × S	S	0	55	0:1	-	-
C × Mj	S × S	S	0	56	0:1	-	-
C × Esp	S × R	R	88	10	15:1	2.61	0.11
U × Est	R × S	R	34	13	3:1	0.18	0.67
U × Mp	R × R	R	-	-	-	-	-
U × OV	R × R	R	55	0	1:0	-	-
U × ON	R × S	R	35	18	3:1	2.27	0.13
U × Mj	R × S	R	47	9	3:1	2.38	0.12
U × Esp	R × R	R	54	0	1:0	-	-
Est × Mp	S × R	R	37	9	3:1	2.38	0.12
Est × OV	S × R	R	44	12	3:1	0.38	0.54
Est × ON	S × S	S	0	52	0:1	-	-
Est × Mj	S × S	S	0	56	0:1	-	-
Est × Esp	S × R	R	48	1	15:1	1.48	0.22
Mp × OV	R × R	R	53	0	1:0	-	-
Mp × ON	R × S	R	43	13	3:1	0.09	0.76
Mp × Mj	R × S	R	48	8	3:1	3.43	0.06
Mp × Esp	R × R	R	52	0	1:0	-	-
OV × ON	R × S	R	39	17	3:1	0.86	0.35
OV × Mj	R × S	R	46	10	3:1	1.52	0.22
OV × Esp	R × R	R	54	0	1:0	-	-
ON × Mj	S × S	S	0	54	0:1	-	-
ON × Esp	S × R	R	50	5	15:1	0.76	0.38
Mj × Esp	S × R	R	53	1	15:1	1.78	0.18

[a]V = Valente; C = Cometa; U = União; Est = Estilo; Mp = Madrepérola; OV = Ouro Vermelho; ON = Ouro Negro; Mj = Majestoso; Esp = Esplendor

Table D. Reaction of parents and F_1 offspring, and expected ratio of resistant (R) and susceptible plants (S) in F_2 generation in each cross inoculated with strain Cl 1614 (65.201)

Crosses	Reaction	Generation				χ^2	*p- value*
		F_1	F_2				
			Obs. Freq.		Expec. Freq.		
			R	S			
V × C[a]	R × R	R	51	0	1:0	-	-
V × U	R × S	R	42	11	3:1	0.51	0.48
V × Est	R × S	R	45	9	3:1	2.00	0.16
V × Mp	R × S	R	45	9	3:1	2.00	0.16
V × OV	R × R	R	51	0	1:0	-	-
V × ON	R × R	R	54	0	1:0	-	-
V × Mj	R × R	R	52	2	15:1	0.60	0.44
V × Esp	R × R	R	46	3	15:1	0.00	0.97
C × U	R × S	R	28	16	3:1	3.03	0.00
C × Est	R × S	R	36	10	3:1	0.26	0.61
C × Mp	R × S	R	42	11	3:1	0.51	0.48
C × OV	R × R	R	54	0	1:0	-	-
C × ON	R × R	R	51	0	1:0	-	-
C × Mj	R × R	R	51	3	15:1	0.04	0.83
C × Esp	R × R	R	48	5	15:1	0.92	0.34
U × Est	S × S	S	0	46	0:1	-	-
U × Mp	S × S	S	0	51	0:1	-	-
U × OV	S × R	R	35	18	3:1	2.27	0.13
U × ON	S × R	R	37	17	3:1	1.21	0.27
U × Mj	S × R	R	40	10	3:1	0.67	0.41
U × Esp	S × R	R	44	10	3:1	1.21	0.27
Est × Mp	S × S	S	0	47	0:1	-	-
Est × OV	S × R	R	36	9	3:1	0.60	0.44
Est × ON	S × R	R	43	10	3:1	1.06	0.30
Est × Mj	S × R	R	43	9	3:1	1.64	0.20
Est × Esp	S × R	R	45	9	3:1	2.00	0.16
Mp × OV	S × R	R	43	9	3:1	1.64	0.20
Mp × ON	S × R	R	39	11	3:1	0.24	0.62
Mp × Mj	S × R	R	43	8	3:1	2.36	0.12
Mp × Esp	S × R	R	42	11	3:1	0.51	0.48
OV × ON	R × R	R	47	0	1:0	-	-
OV × Mj	R × R	R	85	9	15:1	1.77	0.18
OV × Esp	R × R	R	51	3	15:1	0.04	0.83
ON × Mj	R × R	R	47	6	15:1	2.33	0.13
ON × Esp	R × R	R	47	5	15:1	1.00	0.32
Mj × Esp	R × R	R	54	0	1:0	-	-

[a]V = Valente; C = Cometa; U = União; Est = Estilo; Mp = Madrepérola; OV = Ouro Vermelho; ON = Ouro Negro; Mj = Majestoso; Esp = Esplendor

Table E. Reaction of parents and F_1 offspring, and expected ratio of resistant (R) and susceptible plants (S) in F_2 generation in each cross inoculated with strain Cl 1532 (65.152)

Crosses	Reaction	Generation				χ^2	*p- value*
			F_2				
		F_1	Obs. Freq.		Expec. Freq.		
			R	S			
V × C[a]	S × R	R	42	12	3:1	0.22	0.64
V × U	S × S	S	0	55	0:1	-	-
V × Est	S × R	R	36	19	3:1	2.67	0.10
V × Mp	S × R	R	31	15	3:1	1.42	0.23
V × OV	S × S	S	0	54	0:1	-	-
V × ON	S × R	R	30	13	3:1	0.00	1.00
V × Mj	S × R	R	37	17	3:1	1.21	0.27
V × Esp	S × R	R	46	8	3:1	2.99	0.08
C × U	R × S	R	33	14	3:1	0.57	0.45
C × Est	R × R	R	54	0	1:0	-	-
C × Mp	R × R	R	49	5	15:1	0.83	0.36
C × OV	R × S	R	36	7	3:1	1.41	0.23
C × ON	R × R	R	53	0	1:0	-	-
C × Mj	R × R	R	48	4	15:1	0.18	0.67
C × Esp	R × R	R	52	0	1:0	-	-
U × Est	S × R	R	39	13	3:1	0.00	1.00
U × Mp	S × R	R	35	15	3:1	0.67	0.41
U × OV	S × S	S	0	53	0:1	-	-
U × ON	S × R	R	35	15	3:1	0.67	0.41
U × Mj	S × R	R	42	12	3:1	0.22	0.64
U × Esp	S × R	R	71	27	3:1	0.34	0.56
Est × Mp	R × R	R	49	5	15:1	0.83	0.36
Est × OV	R × S	R	42	12	3:1	0.22	0.64
Est × ON	R × R	R	54	0	1:0	-	-
Est × Mj	R × R	R	45	5	15:1	1.20	0.27
Est × Esp	R × R	R	52	0	1:0	-	-
Mp × OV	R × S	R	36	13	3:1	0.06	0.80
Mp × ON	R × R	R	49	4	15:1	0.15	0.70
Mp × Mj	R × R	R	54	0	1:0	-	-
Mp × Esp	R × R	R	50	2	15:1	0.51	0.47
OV × ON	S × R	R	36	15	3:1	0.53	0.47
OV × Mj	S × R	R	42	12	3:1	0.22	0.64
OV × Esp	S × R	R	45	9	3:1	2.00	0.16
ON × Mj	R × R	R	81	9	15:1	2.16	0.14
ON × Esp	R × R	R	54	0	1:0	-	-
Mj × Esp	R × R	R	52	2	15:1	0.60	0.44

[a]V = Valente; C = Cometa; U = União; Est = Estilo; Mp = Madrepérola; OV = Ouro Vermelho; ON = Ouro Negro; Mj = Majestoso; Esp = Esplendor

Table F. Reaction of parents and F_1 offspring, and expected ratio of resistant (R) and susceptible plants (S) in F_2 generation in each cross inoculated with strain LV 238 (65.218)

Crosses	Reaction	Generation				χ^2	*p-value*
		F_1	F_2				
			Obs. Freq.		Expec. Freq.		
			R	S			
V × C[a]	S × R	R	41	15	3:1	0.09	0.76
V × U	S × S	S	0	54	0:1	-	-
V × Est	S × R	R	41	13	3:1	0.02	0.88
V × Mp	S × S	S	0	53	0:1	-	-
V × OV	S × S	S	0	54	0:1	-	-
V × ON	S × R	R	43	11	3:1	0.62	0.43
V × Mj	S × S	S	0	53	0:1	-	-
V × Esp	S × R	R	45	8	3:1	2.77	0.10
C × U	R × S	R	36	18	3:1	2.00	0.16
C × Est	R × R	R	54	0	1:0	-	-
C × Mp	R × S	R	40	14	3:1	0.02	0.88
C × OV	R × S	R	45	8	3:1	2.77	0.10
C × ON	R × R	R	45	6	15:1	2.65	0.10
C × Mj	R × S	R	42	12	3:1	0.22	0.64
C × Esp	R × R	R	51	0	1:0	-	-
U × Est	S × R	R	45	11	3:1	0.86	0.35
U × Mp	S × S	S	0	46	0:1	-	-
U × OV	S × S	S	0	44	0:1	-	-
U × ON	S × R	R	35	16	3:1	1.10	0.29
U × Mj	S × S	S	0	49	0:1	-	-
U × Esp	S × R	R	42	10	3:1	0.92	0.34
Est × Mp	R × S	R	45	9	3:1	2.00	0.16
Est × OV	R × S	R	40	14	3:1	0.02	0.88
Est × ON	R × R	R	96	2	15:1	2.96	0.09
Est × Mj	R × S	R	43	9	3:1	1.64	0.20
Est × Esp	R × R	R	54	0	1:0	-	-
Mp × OV	S × S	S	0	52	0:1	-	-
Mp × ON	S × R	R	42	9	3:1	1.47	0.23
Mp × Mj	S × S	S	0	47	0:1	-	-
Mp × Esp	S × R	R	44	10	3:1	1.21	0.27
OV × ON	S × R	R	45	8	3:1	2.77	0.10
OV × Mj	S × S	S	0	54	0:1	-	-
OV × Esp	S × R	R	44	8	3:1	2.56	0.11
ON × Mj	R × S	R	42	11	3:1	0.51	0.48
ON × Esp	R × R	R	53	3	15:1	0.08	0.78
Mj × Esp	S × R	R	51	3	15:1	0.04	0.83

[a]V = Valente; C = Cometa; U = União; Est = Estilo; Mp = Madrepérola; OV = Ouro Vermelho; ON = Ouro Negro; Mj = Majestoso; Esp = Esplendor
